# Supplementary material for: Global hypo-methylation in a proportion of glioblastoma enriched for an astrocytic signature is associated with increased invasion and altered immune landscape
Source: eLife. 2022 Nov 22;11:e77335. doi: 10.7554/eLife.77335 (PMC9681209; doi:10.7554/eLife.77335)
Supplement: Figure 2—source data 1. [file elife-77335-fig2-data1.zip › Figure_2_source_data_1/Figure_2B/homerResults/motif12.info.html]

Motif 12

## Information for 3-CAGAGAGGCC (Motif 12)

A
G
T
C
C
G
T
A
A
C
T
G
C
G
T
A
A
C
T
G
C
G
T
A
A
C
T
G
A
C
T
G
A
G
T
C
A
G
T
C
  
Reverse Opposite:
